# Osteogenic Differentiation of Human Mesenchymal Stem cells in a 3D Woven Scaffold

**DOI:** 10.1038/s41598-018-28699-x

**Published:** 2018-07-11

**Authors:** Maria Persson, Petri P. Lehenkari, Lena Berglin, Sanna Turunen, Mikko A. J. Finnilä, Juha Risteli, Mikael Skrifvars, Juha Tuukkanen

**Affiliations:** 1 Institute of Cancer and Translational Medicine, Department of Anatomy and Cell Biology, University of Oulu, FI-90014 Oulu, Finland; 2 Medical Research Center Oulu, FI-90014 Oulu, Finland; 3 Department of Textile Technology, Faculty of Textiles, Engineering and Business, University of Borås, S-501 90 Borås, Sweden; 4 Research Unit of Medical Imaging, Physics and Technology, University of Oulu, FI-90014 Oulu, Finland; 5 Department of Applied Physics, Faculty of Science and Forestry, University of Eastern Finland, P.O. Box 1627, FI-70211 Kuopio, Finland; 6 Institute of Cancer and Translational Medicine, Department of Clinical Chemistry, University of Oulu, FI-, 90014 Oulu, Finland; 7 Northern Finland Laboratory Center NordLab University Hospital, Oulu, Finland

## Abstract

Fiber-based scaffolds produced by textile manufacturing technology offer versatile materials for tissue engineering applications since a wide range of crucial scaffold parameters, including porosity, pore size and interconnectivity, can be accurately controlled using 3D weaving. In this study, we developed a weavable, bioactive biodegradable composite fiber from poly (lactic acid) (PLA) and hydroxyapatite powder by melt spinning. Subsequently, scaffolds of these fibers were fabricated by 3D weaving. The differentiation of human mesenchymal stem cells (hMSCs) *in vitro* was studied on the 3D scaffolds and compared with differentiation on 2D substrates having the same material composition. Our data showed that the 3D woven scaffolds have a major impact on hMSCs proliferation and activation. The 3D architecture supports the differentiation of the hMSCs into osteoblast cells and enhances the production of mineralized bone matrix. The present study further confirms that a 3D scaffold promotes hMSCs differentiation into the osteoblast–lineage and bone mineralization.

## Introduction

The major challenge in tissue engineering is to design an ideal scaffold that mimics the three-dimensional (3D) architecture and intrinsic properties of natural tissues or organs. Despite significant efforts in the field, the design requirements for various tissue engineering scaffolds have still not been defined precisely. The pore sizes, together with the porosity, are known to play crucial roles in regulating the morphology and behavior of different cell types^1–3^. The pore sizes required by various cell types differ, and usually pore sizes of several 100 µm are necessary for efficient cell growth, migration and nutrient flow. However, large pore sizes decrease the surface area, limit cell adhesion and prevent the formation of cellular bridges across the structure^4^. Large pores also diminish the mechanical properties of the scaffold due to increased void volume, which is another critical parameter in scaffold design^5^. For scaffolds intended to be used for bone regeneration it has been reported that a pore size in the range of 150–400 µm is optimal to promote bone formation and vascularization within the scaffold^2,3,6^. However, it should be noted that the optimal pore size range is also influenced by the material of the scaffold, its size, as well as vascularization of the surrounding tissues^6^.

Several methods and materials have been applied in combination with multidisciplinary approaches to find the optimal design for the biofabrication of 3D porous scaffold systems for tissue engineering applications^7,8^. Among these processing techniques are methods such as solvent casting, and particulate leaching, gas foaming, emulsion freeze-drying, thermally induced phase separation and rapid prototyping. 3D printing has aroused interest since it is a direct computerized “layer by layer” method to manufacture scaffolds with designed shape and porosity. A major challenge for these techniques is to simultaneously optimize the mechanical properties with an adequate porosity and they still present low reproducibility in combination with high costs^9,10^. For these reasons, far too little attention has been paid to micro-fiber and textile technologies. The human body has various natural fiber structures, mainly collagens within the connective tissue. Muscles, tendons and nerves are also fibrous in nature and therefore cells are used to fibrous structures^11^. Electrospinning, a biofabrication technique capable of producing fibers in the submicro- and nanoscale range, has been widely studied and used in the design of TE scaffolds^4,12^. However, the small fiber diameter in the submicro-and nanoscale range results in low porosity and small pore size, which greatly limits cell infiltration and cell migration through the thickness of the scaffold. When implanted into the body, such electrospun scaffolds will likely loosen over time, which requires re-surgery. In this regard, micro-fibers processed with textile manufacturing technology such as knitting, braiding, weaving or nonwoven can be considered as a potential solution for the biofabrication of complex scaffolds for tissue engineering applications. Such technologies indeed present superior control over the design, manufacturing precision and reproducibility^13^. In addition, the scaffold can further be influenced on a hierarchical level by altering the chemical and/or mechanical properties of the fibers^14,15^.

Using such an approach, Moutos *et al*. reported in 2007 a breakthrough in the scaffold design for the functional tissue engineering of cartilage. They used textile technology to design a biomimetic 3D woven composite scaffold that exhibits and maintains properties similar to those of cartilage^14^. Since then a number of successful studies have been reported using textile technologies to develop scaffolds for different biological tissues^12,13,16–25^. Clearly, the use of textile technology has much to contribute to the development of scaffolds for tissue engineering applications. However, to date, no textile structure with outstanding potential has been used for bone regeneration, although the treatment of injured/fractured bone can be considered economically as one of the most important surgical interventions^26,27^.

For this reason, the objective of this study was to fabricate a fiber-based scaffold utilizing 3D weaving from poly(lactic acid) (PLA) or bioactive PLA-hydroxyapatite (PLA/HA) composite fibers and evaluate its performance as a bone growth platform *in vitro* using bone marrow derived human mesenchymal stem cells (hMSCs). Weaving was selected as a suitable technique, since woven structures are generally stronger and stiffer than nonwoven- or knitted structures. A woven scaffold has therefore greater potential to maintain structural integrity during biomechanical loading^28^. To permit a more precise investigation of the effect of the 3D woven structural architecture on the osteogenic capacity of hMSCs, the study also included 2D substrates using the same material as described in previous studies^29,30^. We hypothesized that a 3D woven scaffold could provide an optimal template to support bone growth.

## Results

### Characterization of the Scaffolds

**Figure 1:**
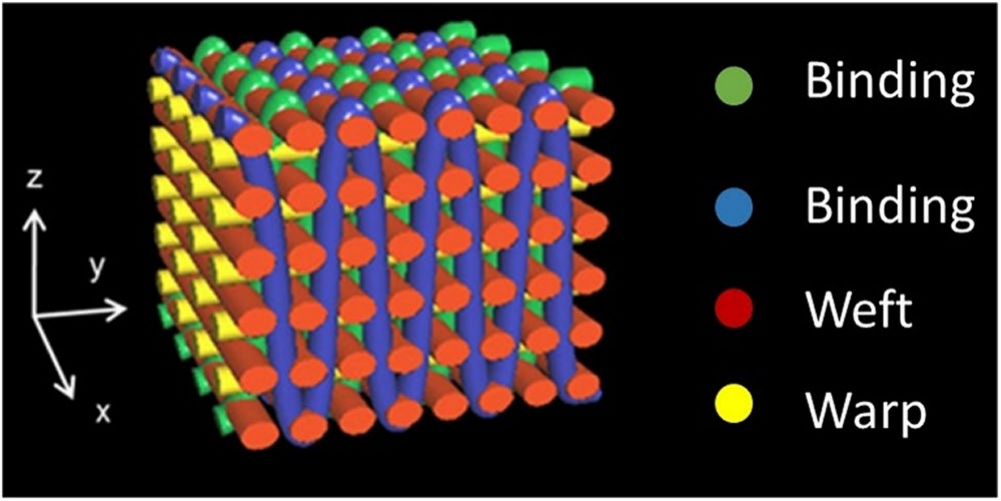
Schematic view of the fiber architecture of a 3D orthogonal woven scaffold and the monofilament yarns are colour coded.

The porosity and the pore-sizes of the 3D woven scaffolds were evaluated using microCT (Fig. 1b). The mean porosity for the PLA 3D woven scaffolds was 64.2% with pore sizes of 224 µm, and a surface area – to - volume ratio of 35.8 mm^−1^. The PLA/HA composite 3D woven scaffolds had a mean porosity of 65.2% with pore sizes of 249 µm and a surface area – to - volume ratio of 34.8 mm^−1^. In addition, the microCT imaging showed good reproducibility of the internal architecture. The thickness for both PLA and PLA/HA composite structures was 2.4 mm. The 2D substrates were 13 mm in diameter and 200 µm thick having surface to volume ratio 5 mm^−1^.

### Characterization of hMSCs

We used cells obtained from only one donor patient (male, 38 years old). Analysis of surface antigens by FACS demonstrated that more than 96% of the cells expressed the surface antigens CD90, CD73, CD105 and CD49e. In addition, the expression of CD34, CD14, CD19, CD45 and HLA-DR was less than 2%, which fulfilled the minimum criteria for cell surface antigens.

### Cell Proliferation and Viability

**Figure 2:**
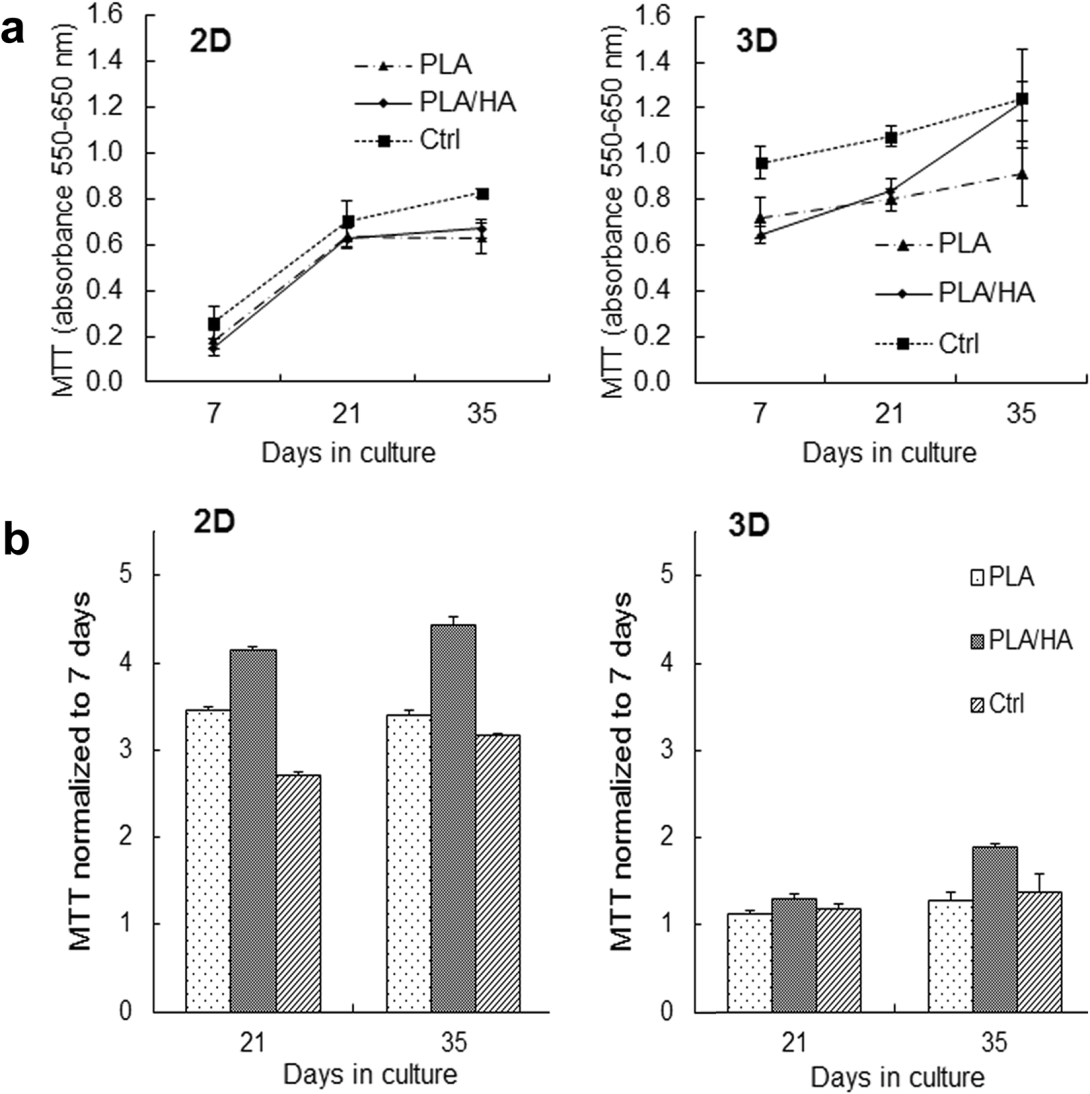
(**a**) MTT activity of hMSCs at 7, 21 and 35 days of culture on 2D substrates (left) and 3D woven scaffolds (right). (**b**) MTT activity of hMSCs normalized to 7 days. Data represent mean ± SD; (n = 4).

The MTT activity results demonstrated an increase in terms of the cells’ metabolic activity after 21 and 35 days of culture in BM compared with the 7-day time point (Fig. 2). This indicates that the materials tested (i.e., PLA and PLA/HA composites) were non-cytotoxic and able to support attachment, proliferation and viability of hMSCs. In addition, it was observed that the MTT activity was higher in cells cultured on substrates containing HA. The positive effect of HA was especially significant in cells cultured on the 2D substrates, reflected by increasing MTT activity over the culture period. This phenomenon was not as clear on the 3D woven scaffolds, although the presence of HA in the composite fiber seems to favor cell proliferation and viability. After normalizing the MTT results with the 7-day results, (Fig. 2b) it was clear that more cellular proliferation occurred on the 2D substrates compared with the 3D woven scaffolds. This could be a result of the initial lower cell density used on the 2D substrate (i.e., 1 500 vs. 50 000 cells/well), which provided more surface area for cell proliferation before reaching confluency. However, the cells seeded on the 3D woven scaffolds did not undergo apoptosis as the MTT activity continued to increase from 21 to 35 days (Fig. 2b). This indicates that the hMSCs cultured on the 3D woven scaffolds had differentiated, as during differentiation cell proliferation usually decreases.

### Cell Attachment and Spreading

**Figure 3:**
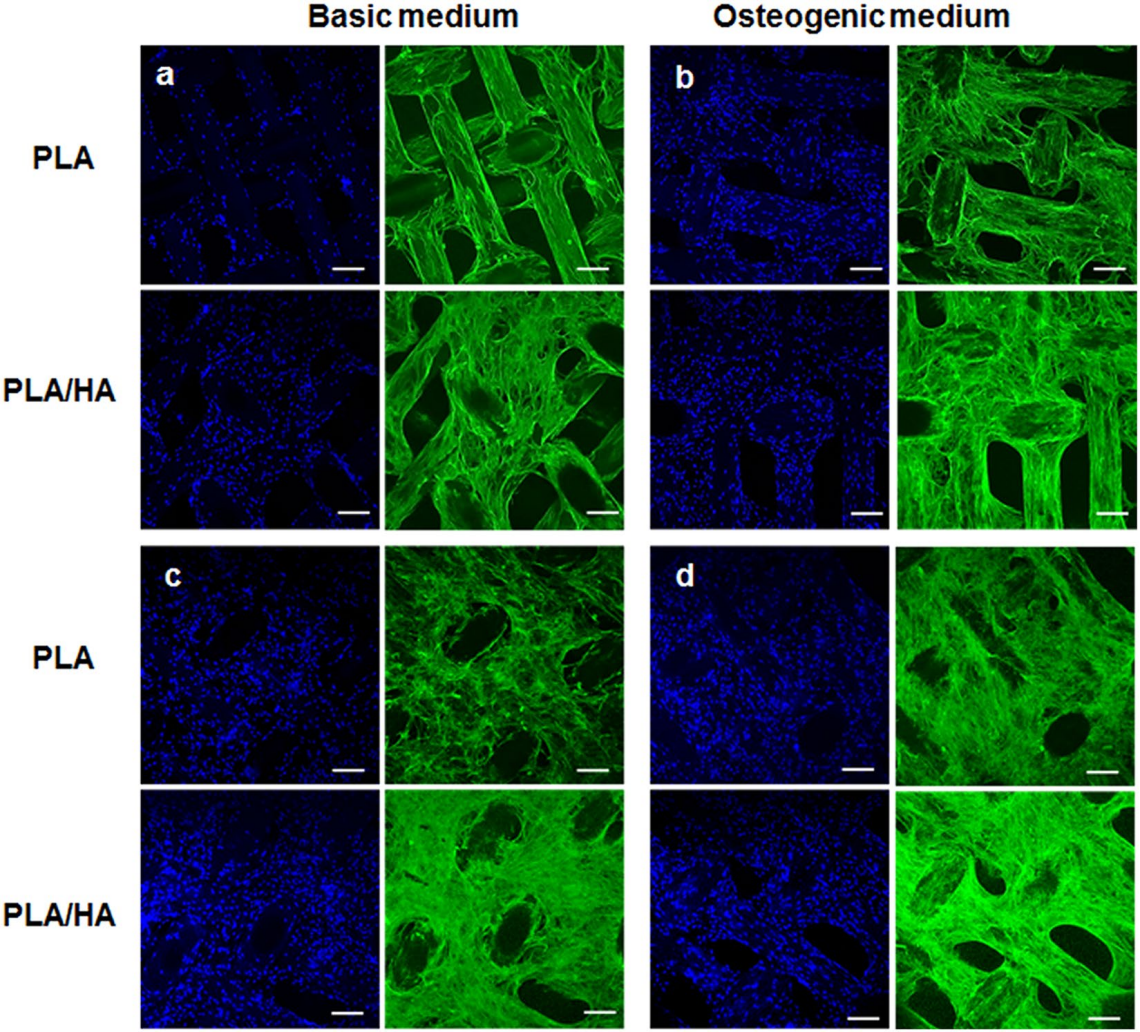
Morphology of attached hMSCs on 3D woven scaffolds made of PLA or PLA/HA composite fibers after 21 days (**a** and **b**) and 35 days (**c** and **d**) as visualized by fluorescent staining of nuclei (blue) and actin filaments (green). The scaffolds were imaged with serial optical sections of confocal microscope from both sides of the scaffold in the rage of the scanning depth. The images are representative single optical sections. Bar = 200 µm.

The 3D woven scaffolds acted as excellent cell support structures over the culture period, as shown in the fluorescence images (Fig. 3a–d). The fluorescence images revealed that hMSCs spread well on all 3D woven scaffolds tested in both BM and OS medium. A slight promotive effect of the presence of HA in the PLA/HA composite 3D woven scaffolds could also be observed on cells cultured in BM at day 21 (Fig. 3a). Cells cultured in BM for 21 days spread better and had begun to bridge across the scaffold’s fibers versus cells cultured on the PLA 3D woven scaffolds. In contrast, this difference was not observed between the PLA and PLA/HA composite 3D woven scaffolds when the hMSCs were cultured in OS for 21 days (Fig. 3b). There the commencement of cell bridging and cell sheet formation across the fibers was observed, regardless of materials. With prolonged culture, (i.e., 35 days) in BM, a dense and continuous cell sheet was prominent and no significant difference between the PLA and PLA/HA composite 3D woven scaffolds could be visualized from the fluorescence images (Fig. 3c,d).

The fluorescent images of hMSCs cultured on the 2D substrate after 21 days demonstrated similar responses regardless of culture conditions, i.e., BM or OS medium. The cells had attached, proliferated and formed a cell sheet that covered the entire surface. No differences in cell shape, which clearly resembled their characteristic fibroblastic morphology, could be visualized between the materials (i.e., PLA and PLA/HA composite 2D substrates, data not shown). A dense and continuous layer of the cells could also be seen after 35 days in BM on both PLA and PLA/HA composite substrates (Supplementary data, Fig. S1a,c). In clear contrast, the visible cells cultured on the 2D PLA/HA composite substrates had decreased sharply compared with the 2D PLA substrates in OS conditions after 35 days (Supplementary data, Fig. S1b,d). On these substrates, the actin stress fibers were not so prominent, indicating that cells had differentiated into osteoblasts similar to those cultured on the PLA/HA composite 3D woven scaffolds.

### Cell Differentiation

**Figure 4:**
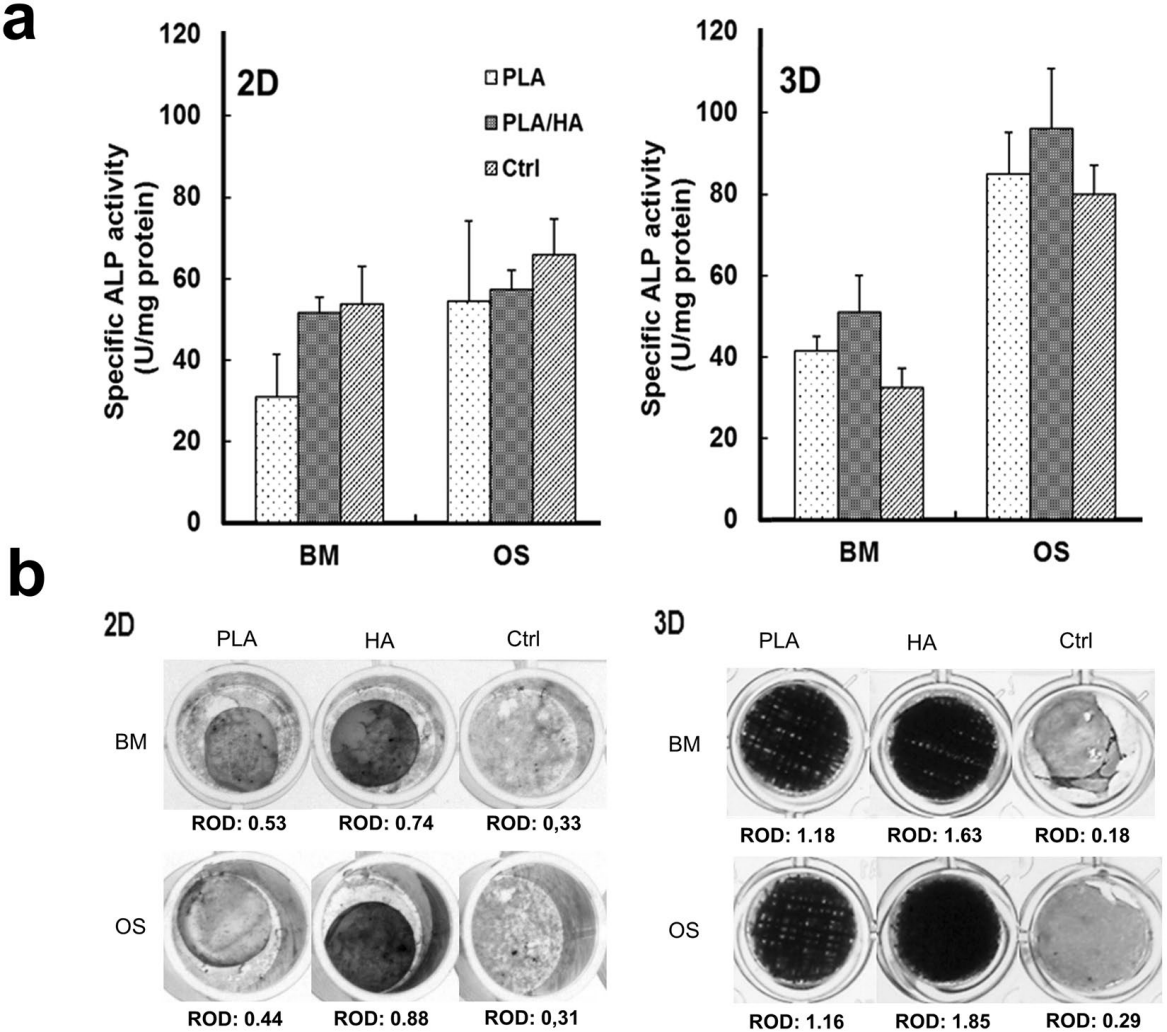
(**a**) Alkaline phosphatase (ALP) activity of hMSCs after 21 days of culture in basal medium (BM) and osteogenic medium (OS) on 2D substrates or 3D woven scaffolds as well as control samples. Initial cell density used in 2D samples was 1.5 × 10^3^ cells/sample and for the 3D woven scaffolds it was 5 × 10^4^ cells/sample. Error bars: mean ± SD. (**b**) Qualitative visualization of ALP activity by histochemical staining after 21 days, representative images are shown (**b**) 2D substrates and 3D woven scaffolds. ROD (relative optical density) is only comparable with the same material within the group.

ALP activity (indicative of osteoblast phenotype) of hMSCs cultured in both BM and OS medium was determined at day 21 (Fig. 4a). As expected, a gradual and significant increase in the ALP levels were found in the hMSCs cultured under OS conditions versus cells cultures in BM, regardless of the substrate configurations. However, a clear increase in the ALP activity was observed in cells cultured on the 3D woven scaffolds compared with those cultured on the same material on the 2D substrate. In addition, the ALP activity of hMSCs cultured in BM on 3D scaffolds containing PLA/HA was also up-regulated (i.e., a fold change increase of 1.35 and 2.6 compared to 3D woven scaffolds made from PLA fibers and ctrl samples, respectively). These results indicate that the PLA/HA composite fibers had an osteoinductive effect on the hMSCs. In contrast, this was not observed in cells cultured on the 2D substrates containing HA. In fact, the ALP activity was not affected by the material composition in the 2D substrates; only the OS medium elevated the ALP activity.

To further study the ALP activity, the samples were histochemically stained (Fig. 4b). Measurement of the relative optical density (ROD) indicated the ALP activity in cells cultured in OS medium on samples containing HA. The ROD value decreased on the PLA samples cultured in BM, as well as in the ctrl samples cultured with the initial cell density of 1500 cells/well.

**Figure 5:**
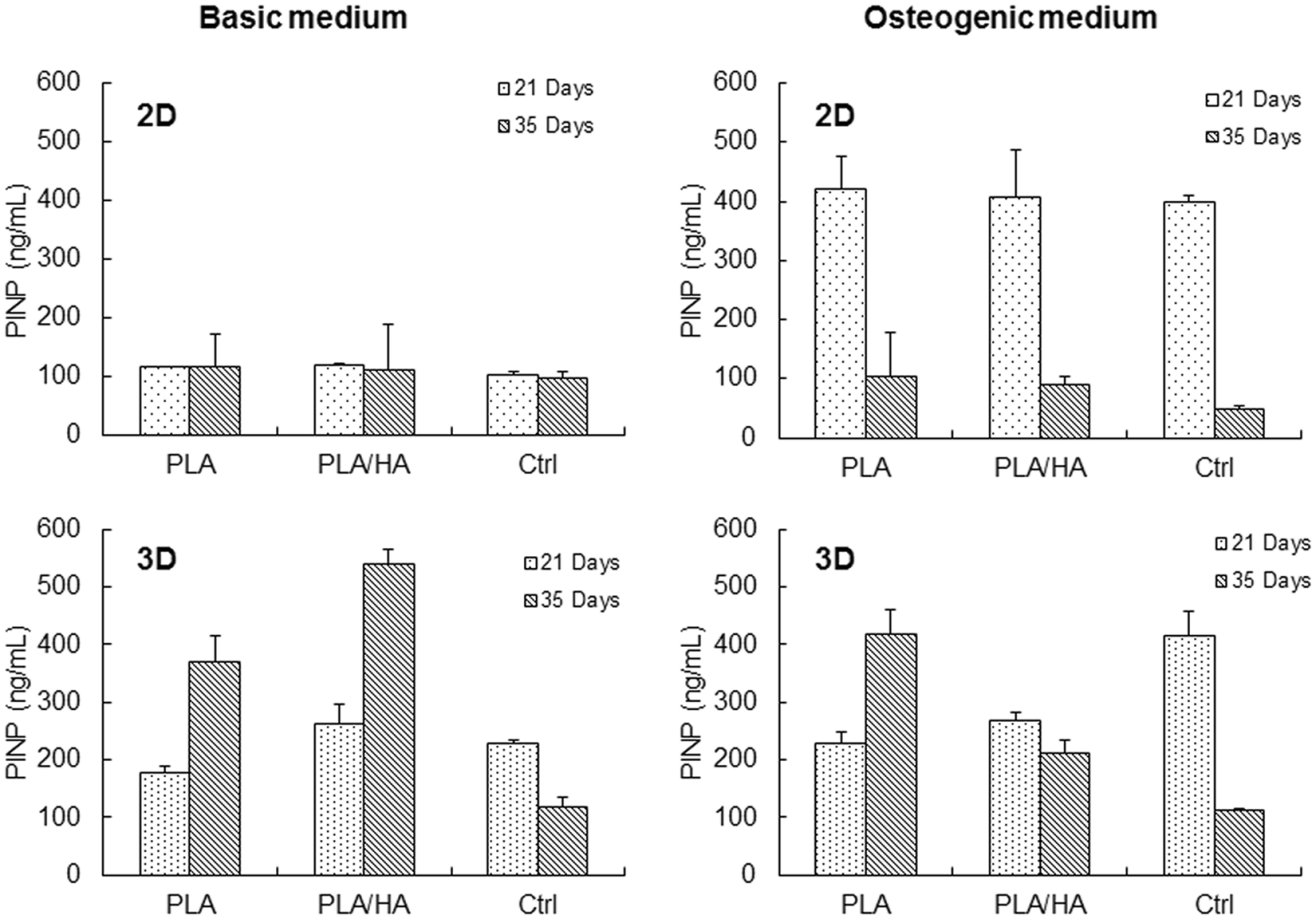
Secretion of PINP by hMSCs cultured for 21 and 35 days in basal medium and in osteogenic medium on various substrates. (Error bars: mean ± SD).

The cells were further characterized for PINP secretion after 21 and 35 days of culture. As shown in Fig. 5, there was no difference between the 2D substrates cultured in the BM at the time points tested. Similar to the ALP activity, cells cultured in OS conditions showed increased PINP levels, which were highest after 21 days. No difference in PINP secretion was observed at the 35-day time point between the 2D samples. For the ctrl samples, it was observed that the cell density had a minor influence when the cells were cultured in BM. PINP secretion in cells cultured in the 3D scaffold under BM conditions was significantly higher compared to their respective 2D sample at all time-points. With prolonged culture (i.e., 35 days) in BM, it was also evident that the 3D scaffolds promote the differentiation of osteoblasts, as confirmed by the increased PINP levels. However, for cells grown on 3D PLA woven scaffolds PINP secretion was not influenced by the culture conditions. The decreased PINP level observed for cells cultured on 3D PLA/HA composite woven scaffolds in OS medium compared to the 3D PLA woven scaffold suggests that the cells cultured on PLA/HA composite scaffolds had already reached their PINP secretion peak by this time point and had differentiated into osteoblast cells. In addition, these results further demonstrate that the cell density in the 2D substrates does not influence the cells potential to differentiate.

### Extracellular Matrix Mineralization

**Figure 6:**
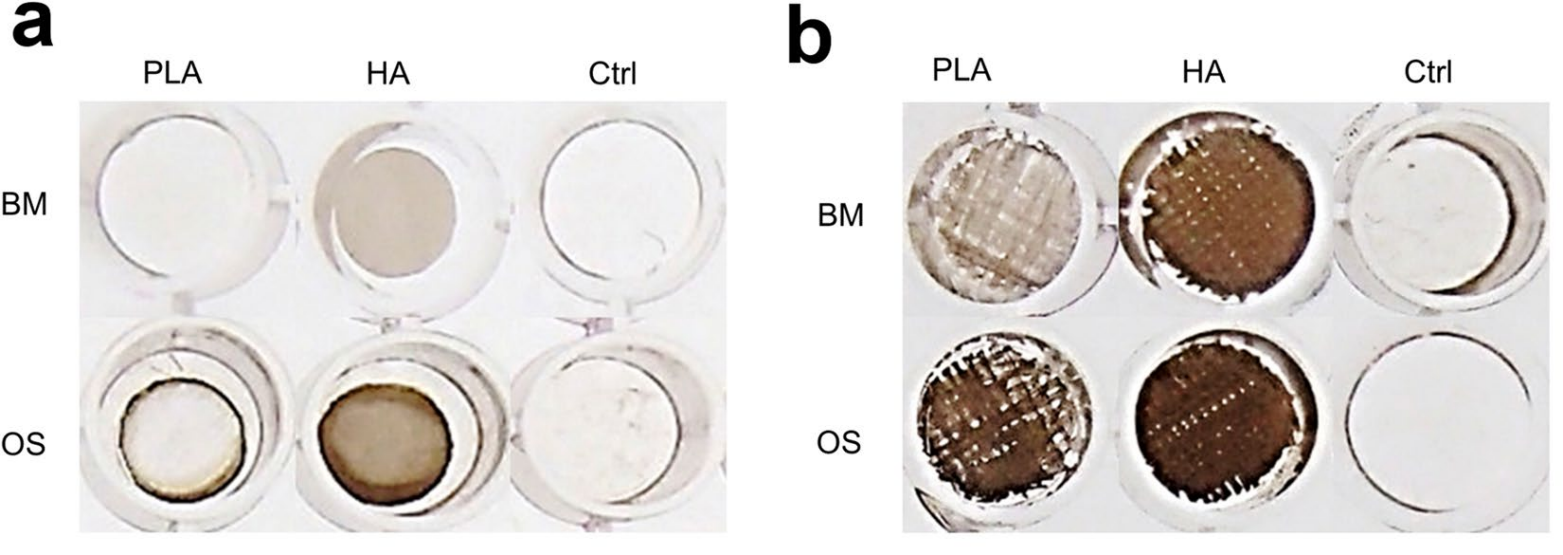
Mineralized nodule formation is illustrated by von Kossa staining after 35 days of culture on (**a**) 2D substrates and (**b**) 3D woven scaffolds. BM = Basal medium, OS = Osteogenic medium.

Visualization of the mineralized matrix deposition within the samples was assessed with von Kossa staining (Fig. 6a,b). The von Kossa staining revealed no mineralized matrix for the 2D substrates cultured in BM, including the ctrl samples. A slight indication of mineralization could be seen in these samples when cultured under OS conditions. However, for the 3D woven scaffolds the von Kossa staining confirmed a clear mineralized matrix, which increased when cultured under OS conditions. In summary, this suggests that the 2D substrate architecture used in this study was not optimal for promoting osteogenesis of hMSCs.

**Figure 7:**
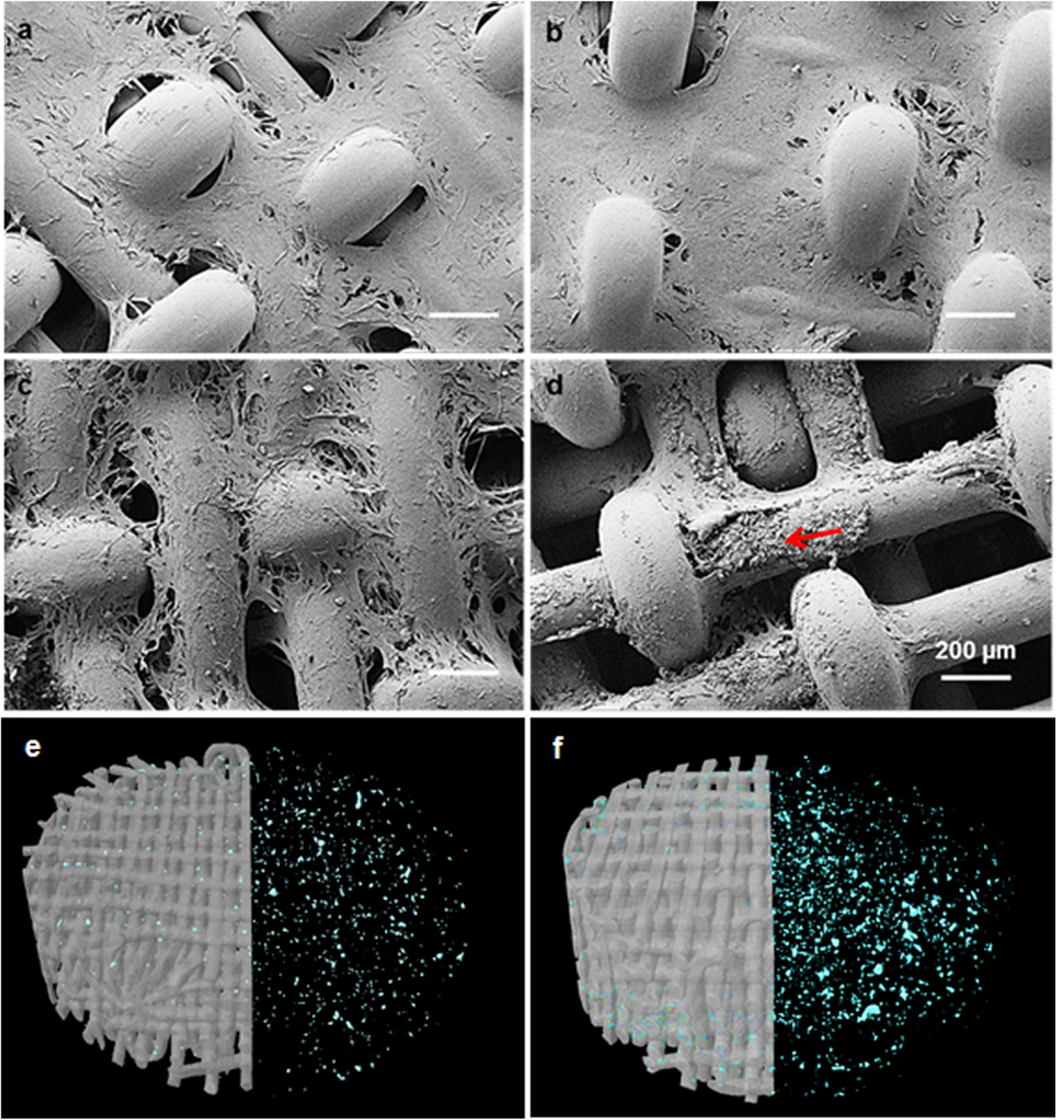
FE-SEM images after 35 days of culture on 3D woven scaffolds made from PLA (**a–c**) and PLA/HA composite (**b,d**) in basal medium (**a,b**) and osteogenic induction medium (**c,d**). Arrow inset in (**d**) = cell mineralization and scale bar: 200 µm. Segmented microCT image of PLA (**e**) and PLA/HA composite (**f**) scaffold shows mineralized nodules through the thickness after 35 days of culture in osteoconductive medium.

Furthermore, the cell morphology and the mineralization of the hMSCs after long-term culture (i.e., 35 days) were analyzed using FE-SEM. During the 35 days of culture in BM, the cells were observed forming a dense cell sheet, which grew through the pores within the 3D woven scaffold, irrespective of the material’s composition, (Fig. 7a,b). Similar to the 3D woven scaffolds, cells were observed to spread uniformly and extensively on the 2D samples, indicating that cells adhered well to the PLA and PLA/HA composite materials ((Supplementary data, Fig. S1)). These results are in good correlation with the observations from the confocal images.

At day 35, FE-SEM confirmed the von Kossa staining that showed the hMSCs had formed a mineralized matrix when cultured on the 3D woven scaffolds. This was especially evident from cells cultured under OS conditions on the 3D woven scaffolds made from the PLA/HA fibers (Fig. 7d). This was not observed for the other samples tested (Fig. 7a–d). The nature of the mineralized phase was further assessed using EDS. The EDS spectra showed that the major elements of the mineralization consisted of oxygen (O), phosphorus (P) and calcium (Ca). In order to confirm that the elements present were related to the cell mineralization, EDS spectra were also taken on human bone slices. The resulting Ca/P atomic ratios were 1.54 and 1.35 for the bone slice and examined mineralization area, respectively (Table 1).

**Table 1:** SEM-EDS analysis for bone slice and cell mineralization on 3D woven scaffold made from PLA/HA fibers.

Element	Bone Slice		Mineralization	
	Weight %	Atomic %	Weight %	Atomic %
Oxygen (O)	48.28	67.74	55.30	72.59
Phosphorus (P)	16.81	12.19	13.12	8.90
Calcium (Ca)	33.57	18.80	22.87	11.99
Others*	1.34	—	8.71	—
Totals	100		100	

*Others: trace elements related to impurities.

Examination of the cells cultured in the 3D PLA woven scaffolds in OS medium (Fig. 7c) indicated that the cells were about to undergo mineralization due to the change in cell morphology as compared with Fig. 7a. The microCT analysis also demonstrated that mineralized nodules were present through the thickness of the scaffold in both PLA and PLA/HA composite 3D woven scaffolds (Fig. 7e,f). This was not observed for uncultured 3D woven scaffolds. The number of mineralized nodules was significantly less in PLA 3D woven scaffolds compared with PLA/HA composites. In addition, it was also visually evident that PLA 3D woven scaffolds had started to degrade, and fibers were beginning to fuse to each other. Taken together, these results clearly demonstrate that differentiation of the hMSC was enhanced in the 3D woven scaffolds and that the matrix mineralization had accelerated in presence of HA in the fibers.

## Discussion

It is well known that the architectural parameters, such as porosity, pore size and interconnected pores as well as the surface properties are important features to consider in scaffold design for bone tissue engineering. All aspects are linked, and modification of one of the parameters typically affects the outcome. In this study, 3D weaving was used to create the scaffolds since this fabrication method allows the development of a scaffold with accurately controlled pore size, porosity and with interconnecting pores. In addition, PLA was used as the scaffold material, since it is FDA approved, degrades to nontoxic products, possesses favorable mechanical properties and is well suited for use as a fiber^29,31^. However, due to PLAs hydrophobic nature, it lacks cell recognition signals and does not offer biological adhesion sites, hence cell response to the material’s surface is poor^32^. Although the protein adsorption on neat PLA is as good as to control TCPS, in comparison the cell attachment on PLA is minor. However, after 24 to 48 h cell culture the osteoblastic cells started to attach on neat PLA indicating that PLA surfaces are modified in culture medium^32^. This seems to be the case also here since longer culture times allow PLA to condition and hMSCs to attach well on them. However, initial cell attachment was achieved already with 5% HA in PLA. Therefore, in order to increase the bioactivity of PLA, HA was incorporated into the PLA matrix to provide a bioactive composite fiber. In addition, HA is a major component of bone matrix and has emerged as the most suitable bioceramic material for bone therapies because of its excellent ability to adsorb proteins^31,33^. Protein adsorption is an important characteristic feature, since that mediates the initial cell response to the material^31,34^. Thus, PLA/HA composites have been proposed to be good candidates for bone substitute materials.

To maintain the favorable bone bonding capacity and bioactivity of the HA particles it is important that they are exposed on the outer surface of the material. This aspect, has been carefully studied previously and the method used here to produce the fibers and films was proven to allow the HA particles to be exposed on the PLA surface. It was demonstrated that the surface morphology, (i.e., topography, chemistry, and particles/area) of the composite fibers were similar to the surfaces of melt extruded films^29,32^. Therefore, the surfaces of the 2D films served as excellent control substrates for the fiber surfaces in this study. In this way the influence of the 3D woven scaffolds architecture on the osteogenic capacity of hMSCs could be studied in detail.

With respect to the role of the 3D woven scaffolds made from PLA and/or PLA/HA composite fibers, it was demonstrated that they showed superior osteogenic potential, compared to the control 2D substrates made from the same material. Thus, it was possible to substantiate a link between the effect of the 3D woven architecture of the scaffold on cell proliferation and differentiation into osteoblasts. The differentiation of osteoprogenitors into osteoblasts is characterized by mineralization, and based on evidence from this study cell mineralization did not occur during the time cells were cultured on the 2D substrates. Hence, it is clear that the cell behavior in 2D and 3D environments is very different and that exposure of hMSCs to 2D substrates may cause loss of function because of a change in cell shape and polarity. The 3D woven scaffolds with a large surface area are, therefore, believed to overcome this, since they better mimic the 3D biological environment of native tissue^35^.

As stated before, the scaffold’s pore-size and porosity are of significant importance for sufficient cell penetration, tissue in-growth and vascularization in order to be successful *in vivo* applications. Generally it has been estimated that *in vivo* cells in metabolically active tissues are within 100 µm of an oxygen source and that the average size of a human osteon is approximately 223 µm^36^ Based on this, it can be hypothesized that the optimal pore size range of a scaffold designed for bone tissue engineering should approximate this value. Therefore, the 3D woven scaffolds used in this study were designed to have a pore-size around 224 µm and 249 µm for the PLA and PLA/HA composite 3D woven scaffolds, respectively.

However, the osteogenic behavior of cells is not only influenced by the geometrical environment, but also by their seeding density. The seeding of the cells in a 3D scaffold is difficult since there is a risk that the cells go through the scaffold and attach on the cell culture plate instead. Bitar *et al*.^37^ demonstrated that the cell seeding density in a 3D scaffold clearly affects the cells’ ability to proliferate and differentiate. Therefore, the cell seeding density used for the 3D woven scaffold, i.e. 5 × 10^4^ cell/scaffold, was optimized in a pilot study based on their confluence before the experiment started (data not shown). In addition, we used the cell seeding density of 1.5 × 10^3^ cells/well for the 96-well plates when investigating the 2D samples. In order to determine whether the seeding density of the hMSCs had an impact on proliferation and differentiation when cultured in a 2D environment, TCPS cell culture dishes were used as a control. However, caution should be taking into account when comparing the control groups as the initial cell density was different and why we also had two control groups of TCPS (for 2D and 3D). The TCPS groups can only be comparable in view of the hMSCS behaviour using different initial cell density (i.e., 15 6250 cells/cm^2^ vs 4690 cells/cm^2^). These controls verified that the experiments had been done successfully.

Based on the MTT test the reduction of the proliferation in 3D scaffolds at 21 and 35 days may indicate also the influence of bulk transfer of cells and therefore an early contact inhibition. It may also indicate cell differentiation, since proliferation is known to be reduced when cells start differentiation.

Another key factor in tissue engineering is the choice of a reliable source of cells that allows isolation, easy expansion to higher passages and the ability to control cell differentiation^38^. hMSCs were selected for this study due to their ability to adhere to cell culture plastic, which makes them easy to culture, and their accessibility endorses their utility for bone tissue engineering approaches. In addition, it has been suggested that hMSCs are an “immune privileged” type of cells, which would make them suitable for allogenic and xenogenic transplantation^39^. Differentiation of the hMSCs into osteoblastic lineages *in vitro* is usually revealed by their capacity to express ALP, as well as type I procollagen^40^. Type I collagen is expressed by osteoblasts during the initial period of proliferation and ECM-synthesis, whereas ALP is expressed during the post proliferation period of ECM maturation. After cellular maturation, the matrix mineralization can be visualized, for instance by von Kossa staining and SEM analysis. According to the results from this study, the cells’ ALP activity significantly increased when cultured on 3D woven scaffolds as compare to the 2D substrates. In addition, it was demonstrated that the ALP activity was induced by the presence of bioactive agents, such as ascorbic acid, β-glycerophosphate and dexamethasone in the OS cell culture medium. From the ctrl samples, it was also demonstrated that, when the bioactive agents were present, a higher cell seeding density resulted in increased ALP activity. These results are in good agreement with previous studies that also demonstrate that the ALP activity is dependent on the cell density^37^, but not on the 2D substrates themselves^41^. This difference in the cell seeding density was less apparent when PINP secretion was measured. In addition, the PINP result strongly indicates that the bioactive agents need to be present in the medium to promote osteogenesis of hMSCS when cultured on 2D substrates. In contrast, the PINP secretion results demonstrated that the 3D woven scaffold itself could cause cell differentiation in BM. Osteogenic medium did not influence PINP secretion in pure PLA 3D scaffolds but in PLA/HA scaffolds PINP was decreased at 35 days’ time point indicating osteogenic differentiation and compromised collagen synthesis in contrast to fibrosis.

However, the single most striking observation to emerge from this study was the matrix mineralization in the 3D woven scaffold made from PLA/HA composite fiber (Fig. 7d and f) when cultured under osteogenic conditions. When compared to the results with the 2D substrates, it is evident that the use of a 3D scaffold is essential for the hMCS to differentiate into osteoblasts and to start to mineralize. In addition, it was clear that the presence of HA accelerated the osteogenesis and mineralization of hMSCs. SEM reveals that the nodule formation is on the surfaces and cannot be mixed with the HA content of the PLA/HA fibers. The nanoparticles <200 nm are below the detection limit of the µCT analysis and may just increase the background of the analysis, which has been taken into account when segmenting the images.

When interpreting these promising data, it should be kept in mind, that in these experimental conditions the implant was not exposed to stress or movement, which might influence the structure of the 3D woven scaffolds. Hence, these data support only use of the 3D woven composite scaffold under stable conditions, for example as an osteoinductive void filled after the removal of tumors, bone cysts or similar intraosseous defects, that are not readily exposed to stress or movements.

## Materials and Methods

### Scaffold Design and Fabrication

PLA (NatureWorks 62011D, Nature Works LLC, MN, USA) and bioactive PLA composite fibers containing 20 wt % HA particles (nanopowder, <200 nm, Sigma-Aldrich Co, St. Louis, MO, USA) were melt-spun into fibers as previously described and characterized in detail^27^. Briefly, the fibers were prepared using a three-step process. The initial step was the preparation of a master batch using a solvent mixture (i.e., 1,4-dioxane) followed by an extrusion step to form PLA/HA pellets. In the third step, monofilament fibers were melt-spun from the extruded pellets. This technique enables homogeneous dispersion of HA in the PLA matrix as well as surfaces with exposed HA particles^29,32^. Fibers with an average diameter of 170 µm were then woven into a 2.4 mm thick porous 3D orthogonal woven structure using a prototype handloom (Atelier ARM, Biglen, Switzerland). The fibers were placed in three mutually orthogonal directions to form a structure consisting of 11 layers; five warp layers (*x*-direction) and six weft layers (*y*-direction), which were bound together by a warp set through-the-thickness (*z*-direction), as shown in Fig. 1a. For comparison, 2D control substrates were also prepared from the same master batch by melt extrusion of the material into a 200 um thick film as described previously^32^. The 3D woven scaffolds and the films were then cut into 6 mm diameter discs using a biopsy punch (Kruuse, Langeskov, Denmark) and the samples were used for further experiments.

### Scaffold Characterization

The porosity, pore-size distribution and surface area of the scaffolds were measured from images collected with a micro-computed tomography system (Skyscan 1272, Bruker microCT, Kontich, Belgium). The source voltage was set to 45 kV and filtered with a 0.25 mm Al filter. After the x-ray source was stabilized, the scaffolds were imaged using a 5 μm isotropic voxel size. Native scaffolds were imaged in air and cell containing scaffolds in phosphate buffered saline (PBS, Sigma-Aldrich Co, St. Louis, MO, USA). The cell-containing scaffolds were imaged for morphology and mineralization nodule quantification after 35 days of cell culture. The images were collected over 360° every 0.3°. Three images were collected for each step and averaged to reduce noise. The images were reconstructed with a program provided by the manufacturer (NRecon, Bruker microCT, Kontich, Belgium) applying suitable ring-artifact and beam hardening corrections. The reconstructed images were analyzed in the manufacturer’s CTAnalyzer program (CTAn, Bruker microCT) and using a global threshold, which made it possible to segment PLA and PLA/HA composite scaffold and mineralization nodules. The 3D pore size in the scaffold was defined with trabecular separation parameter corresponding to fiber separation in woven material which is equal to mean pore size.

### Isolation of Bone Marrow -Derived hMSCs

With approval from the Ethical Committee of the Northern Hospital Districts, Finland, bone marrow derived hMSCs were obtained after informed consent from a 38-year old male donor undergoing a hip replacement operation for posttraumatic osteoarthritis. Primary isolation and culture of hMSCs was performed as described in detail earlier^42^. hMSCs were selected from only one single donor to minimize variation related to different sources and interpretation of the results.

### Flow Cytometric Characterization of hMSCs

The hMSCs were characterized with respect to the minimal criteria panel of surface antigens proposed by the Mesenchymal and Tissue Stem Cell Committee of the International Society for Cellular Therapy, as previously described^43^. hMSCs were detached from the cell culture flask and suspended in PBS with 0.5% bovine serum albumin (BSA). Fluorescence-activated cell sorting (FACS) analysis was performed to characterize surface antigen expression using the following conjugated antibodies: CD90 (FITC, Stem Cell Technologies, Grenoble, France), CD73 (phycoerythrin (PE), BD Biosciences, San Jose, CA), CD105 (FITC, Abcam), and CD49e (PE, BD Bioscience). A negative panel of the following surface antigens were incubated simultaneously as a group in the same sample: HLA-DR (PE, BD Biosciences), CD34 (PE, BD Biosciences), CD45 (PE, BD Biosciences), CD14 (PE, BD Biosciences) and CD19 (PE, BD Biosciences). In addition, the following isotype controls were used: FITC Mouse IgG2a k (BD Biosciences) and PE Mouse IgG2a k (BD Biosciences). The cells were washed after incubation (20 min in RT) with PBS + 0.5% BSA and analyzed using a FACSAria, equipped with laser emission at 488, 633, and 407 nm. The FITC, PE and APC channels were used to detect the emission of the conjugated surface antigens and the data were analyzed using Cyflogic (CyFLo Ltd, Turku, Finland). Cell debris was gated out from all samples, the definition of a positive gate being based on isotype and unlabeled cells.

### Cell Seeding and Differentiation of hMSCs

hMSCs were thawed, plated in a culture flask (T175) and cultured in α-MEM (minimum essential medium; Gibco, Paisley, UK), supplemented with 10% heat-inactivated FBS (Fetal bovine serum, Autogen Bioclear, Wiltshire, UK), 20 mM HEPES (Gibco), 100 U/mL of penicillin, 0.1 mg/mL of streptomycin (Gibco), and 2 mM L-glutamine (denoted as basal medium “BM” hereafter) and incubated at 37 °C in 5% CO_2_ and 95% air. The medium was replaced at a rate of 50% every 3 days until the cells reached 90% confluence, after which they were passaged and replaced into a 2-stack cell culture chamber (Corning, CellSTACK Culture chambers, Sigma).

Prior to the cell studies, the scaffolds were placed in a 96-well tissue-culture polystyrene plate (TCPS) and immersed in 70% ethanol for 1 h, in order to remove potential residues from the sample preparation. The scaffolds were then further washed with 1 × PBS for 3 × 15 min and subsequently immersed in PBS overnight. Finally, the trypsinized hMSCs were seeded in 50 µl on the scaffolds or directly onto the 96-well plate (ctrl) at a density of 5 × 10^4^ cells/well. For the 2D control study, the cell density was 1.5 × 10^3^ cells/well as less surface area was available for the cells. The cells were incubated at 37 °C for 4 h to allow the cells to diffuse into and adhere to the scaffold before the addition of 100 µl of culture medium to each well. Cells used in this study were from the fourth passage (P4), and 50% of the culture media was changed three times a week. For osteogenic differentiation studies, the cells were cultured in the osteogenic induction medium (denoted as “OS” hereafter) containing 0.1 µM dexamethasone, 10 mM sodium β-glycerophosphate, and 0.05 mM ascorbic acid-2-phosphate (Sigma-Aldrich, St. Louis, MO, USA), in addition to the basal cell culture medium as described above. We compared the osteogenic response of the selected clone to other clones in our storage and the induction of ALP activity was at the same range as with most of the cell lines when the cell were cultured in OS instead of BM (Supplementary data, Fig. S2).

### Immunofluorescence Microscopy, Cell Morphology and Viability

After 21 and 35 days hMSCs cultured on the 3D scaffold or the 2D substrates either in BM or OS (*n* = 4) were fixed with 3% paraformaldehyde (PFA) solution containing 2% sucrose for 10 min at room temperature (RT) and rinsed with PBS twice. The cells were then stained with 1:200 diluted FITC-labeled phalloidin (Sigma) for 20 min at 37 °C, followed by 1:800 diluted Hoechst 33258 (Sigma) staining for 10 min at RT. Afterwards, the fluorescent signals were observed using a confocal laser-scanning microscope (Zeiss LSM 780, Oberkochen, Germany). The cell proliferation and cell viability were measured using a MTT (3-(4,5-dimethylthiazol-2-yl)-2,5 diphenyltetrazolium bromide) assay at 7, 21 and 35 days of cell culture in BM. The cell culture medium was completely replaced with new medium containing MTT (0.1 mg/ml medium) and the test samples were incubate at 37 °C for 3 h. Unincorporated dye was removed, dimethyl-sulfoxide (DMSO) was added and absorbance was quantified by plate reader (Victor 2, PerkinElmer Life Science/Wallac Oy, Turku, Finland) at 550 nm. The result was background-corrected at an absorbance of 650 nm. The results were expressed as the average absorbance values of four replicates.

### Alkaline Phosphatase Activity

Specific alkaline phosphatase (ALP) activity was measured after 21 days of culture (*n* = 5). The samples were extracted into an assay buffer containing 50 mM Tris-HCl, 0.1% Triton-X-100 and 0.9% NaCl (pH 7.6), and the lysate was frozen at −70 °C. The lysate samples were then thawed, and enzyme activity was determined using 0.1 mM 4-*p***-**nitrophenylphosphate as a substrate in an assay buffer containing 0.1 M Tris and 1 mM MgCl_2_ (pH 10.0 Sigma-Aldrich). After incubation at room temperature for 30 min, the reaction was stopped by the addition of 0.1 M NaOH, and the absorbance was measured at 405 nm with a plate reader (Victor 2). Five parallel samples were measured in triplicate. The protein content was determined by Bio-Rad Protein Assay (Bio-Rad Laboratories) with bovine serum albumin as standard. The specific ALP activity was calculated as absorbance at 405 nm/protein mg/ml. For ALP histochemical staining (*n* = 4), the cells were washed with PBS, fixed with citrate-acetone formaldehyde fixative, washed with deionized water, and stained for enzyme activity with the alkaline solution containing naphthol AS-BI phosphate and fast red violet LB base according to the manufacturer’s instruction (alkaline phosphate kit, Sigma-Aldrich).

### Procollagen I N-terminal Propeptide Analysis

The amount of synthesis of type I procollagen was quantified from the culture medium after 21 days and 35 days. Single 20 µl samples (*n* = 4) were analyzed using the automated iSYS instrument (Intact Procollagen I N-terminal propeptide analysis (PINP), IDS, Newcastle, UK). The method has been validated using serum samples, where intra-assay and inter-assay coefficients of variation were about 3% and between 4.2–5.3% respectively^44^.

### Extracellular Matrix Mineralization

The cell mineralization was characterized using Zeiss ULTRA Plus field-emission scanning electron microscopy (FE-SEM) (Oberkochen, Germany). The cells cultured for 35 days (*n* = 4) were rinsed twice with PBS and fixed in 2.5% gluteraldehyde for 15 min. The scaffolds were then dehydrated in an ascending ethanol series (30%, 50%, and 70%) for 5 min in each concentration and exposed for critical point drying (BAL-TEC CPD 030). To avoid charging, the samples were coated with a 5 nm-thick platinum layer using an Agar high-resolution sputter coater (model 208HR) before placement in the FE-SEM chamber. For elemental analysis, energy-dispersive X-ray spectroscopy (EDS) was used to confirm the hMSCs mineralization from carbon-coated samples. For comparison, human bone slices were also analyzed using EDS. For von Kossa silver staining of mineralized nodules, the cells were washed with PBS, covered with 1% silver nitrate (AgNO_3_) for 30 min in room temperature and washed with deionized water. In the second step, the samples were covered with 2.5% sodium thiosulfate for 5 min and again washed with deionized water.

### Densitometry

The intensity of ALP staining was quantified by digital densitometry. The samples were imaged on precision light table with digital camera (Micropublisher, QImaging, BC, Canada) equipped with micro Nikkor 55 mm lens (Nikon, Tokyo, Japan) and the data was acquired with MCID Core 7 image analysis system (InterFocus Imaging ltd, UK). The illumination settings were kept constant and mean pixel brightness values were gathered by using constant size of sampling tool over each sample. The gray values were then converted to relative optical densities (ROD = log_10_ (255/Pixel value).

### Statistical analysis

All values are expressed as mean values ± standard deviation (SD). It is well known that the differentiation of hMSCs may vary between different donors. We used only one donor, which does not enable a reliable robust statistical analysis of the data. Also the number of samples is fairly small. For those reasons we have used only graphical and visual approach for the exploratory data analysis (EDA).

## Electronic supplementary material

Supplementary information
