# Supplementary material for: Osteogenic Differentiation of Human Mesenchymal Stem cells in a 3D Woven Scaffold
Source: Sci Rep. 2018 Jul 11;8:10457. doi: 10.1038/s41598-018-28699-x (PMC6041290; doi:10.1038/s41598-018-28699-x)
Supplement: Supplementary file 1 — Supplementary information [file 41598_2018_28699_MOESM1_ESM.docx]

**Supplemental Information for**

**Osteogenic Differentiation of Human Mesenchymal Stem cells in a 3D Woven Scaffold**

Maria Persson ^1,2^, Petri P. Lehenkari ^1,2^, Lena Berglin ^3^, Sanna Turunen ^1^ , Mikko A. J. Finnilä ^4,5^, Juha Risteli ^6, 2^, Mikael Skrifvars ^3^, Juha Tuukkanen ^1,2^

^1 Institute of Cancer and Translational Medicine, Department of Anatomy and Cell Biology, University of Oulu, FI-90014, Oulu, Finland^

^2 Medical Research Center Oulu, FI-90014, Oulu, Finland^

^3 Department of Textile Technology, Faculty of Textiles, Engineering and Business, University of Borås, S-501 90 Borås, Sweden^

^4 Research Unit of Medical Imaging, Physics and Technology, University of Oulu, FI-90014, Oulu, Finland^

^5 Department of Applied Physics, Faculty of Science and Forestry, University of Eastern Finland, P.O. Box 1627, FI-70211, Kuopio, Finland^

^6 Institute of Cancer and Translational Medicine, Department of Clinical Chemistry, University of Oulu, FI-90014, Oulu, Finland and Northern Finland Laboratory Center NordLab University Hospital, Oulu, Finland^

**
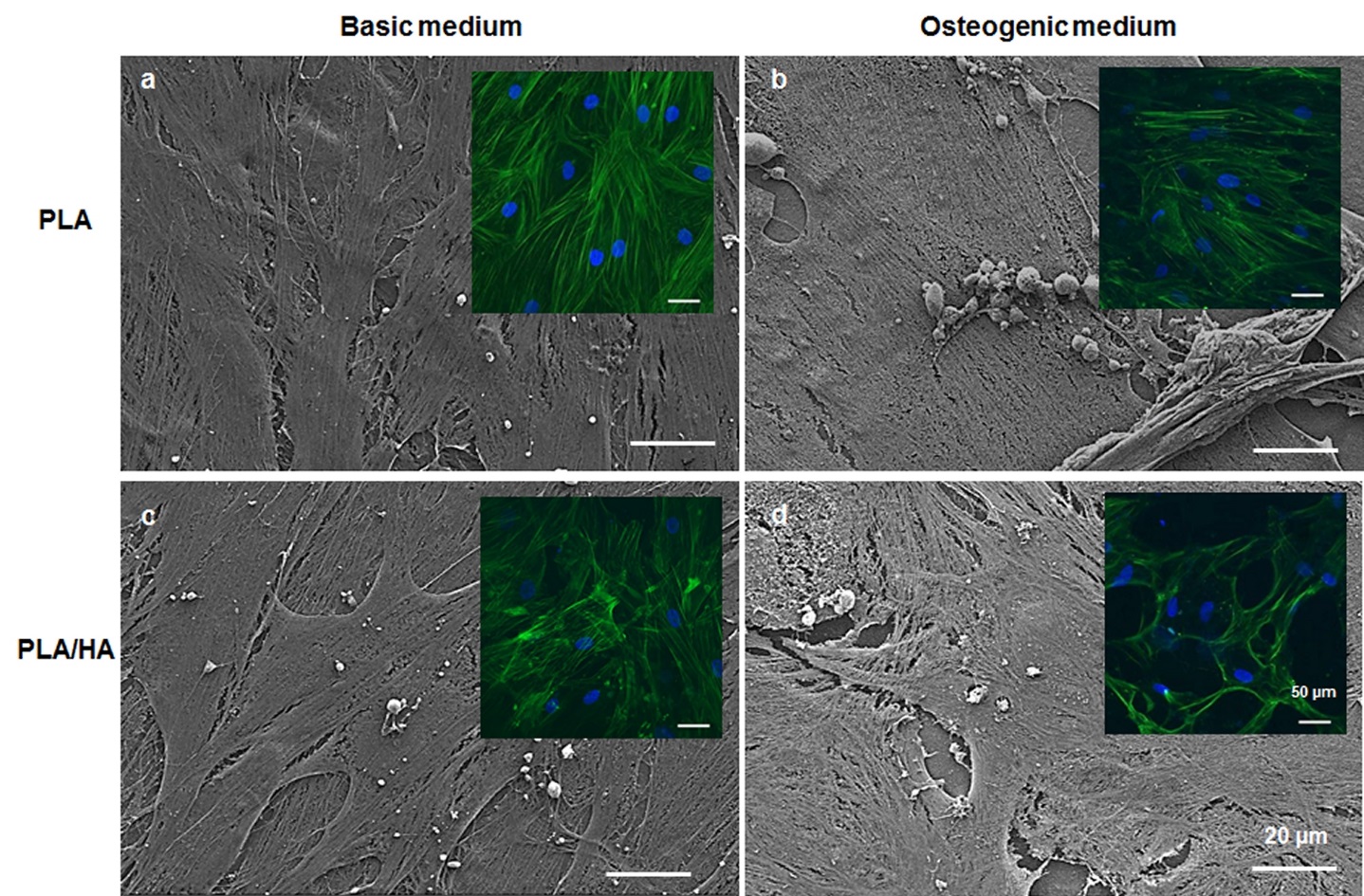
**

**Figure S1.** FE-SEM images of hMSCs after 35 days of culture on 2D substrates of PLA (a and b) and PLA/HA composites (c and d). Figure insets represent the confocal images of the cells at the same time point. Scale bar for SEM = 20 µm and for inset = 50 µm.


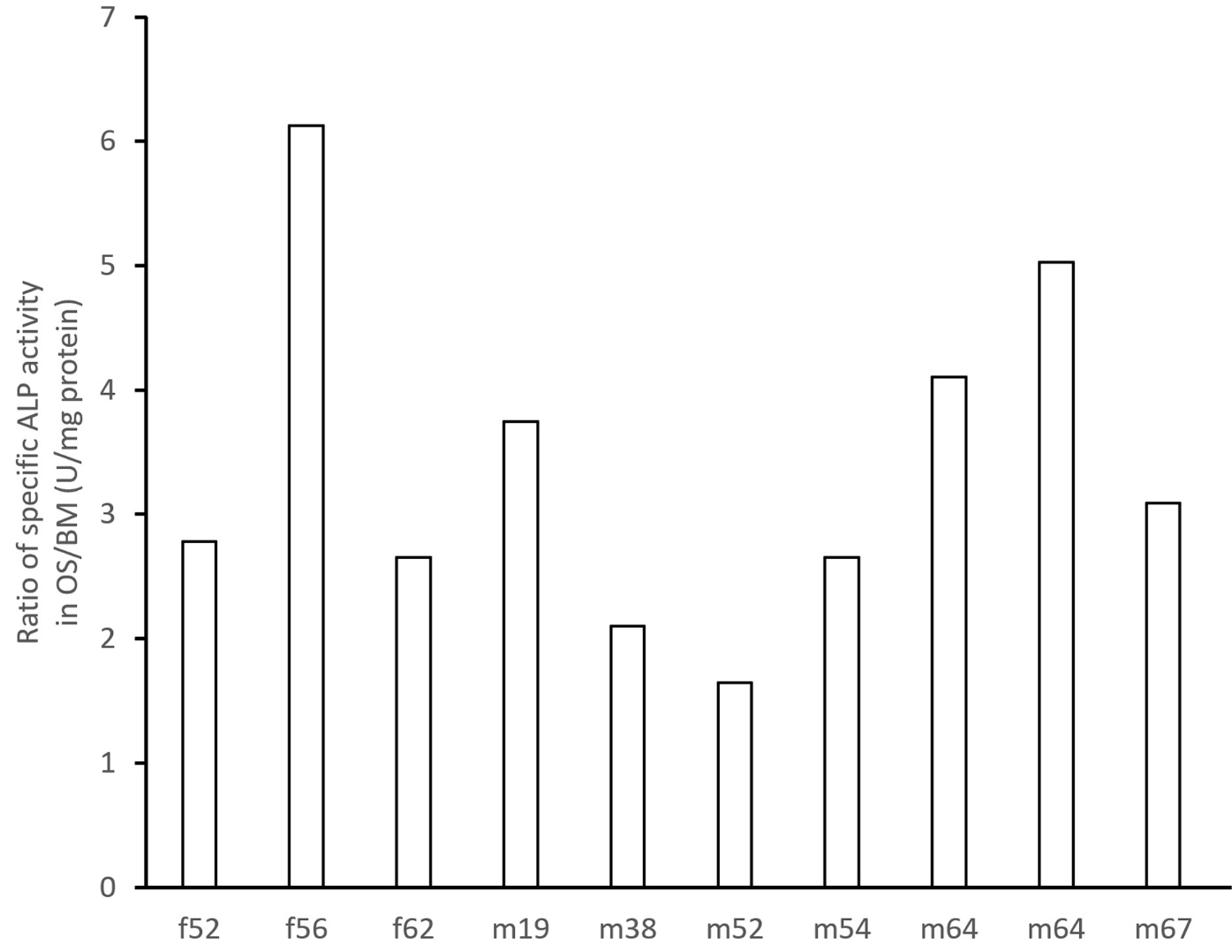


**Figure S2.** Ratio of the alkaline phosphatase (ALP) activity (units per mg protein) of mesenchymal stem cells cultured 21 days in osteogenic medium (OS) compared to culture of same time in basal medium (BM). The donor gender (f=female, m=male) and age are given. The donor for the cell line used in the current paper was 38 years old male (m38).
